# A Green Method for the Determination of Cadmium in Natural Waters Based on Multi-Fibre Supported Liquid Membranes

**DOI:** 10.3390/membranes13030327

**Published:** 2023-03-12

**Authors:** Juan J. Pinto, Victoria Mánuel, Carlos Moreno

**Affiliations:** Department of Analytical Chemistry, Faculty of Marine and Environmental Sciences, University of Cadiz, 11510 Puerto Real, Spain

**Keywords:** hollow fibre liquid-phase microextraction, liquid membrane, cadmium, Cyanex^®^ 272, preconcentration, natural waters

## Abstract

Supported liquid membranes have been used to implement a hollow fibre liquid-phase microextraction (HF-LPME) method for the preconcentration of Cd(II) in natural waters as a sample preparation step for its determination by high-resolution continuum source graphite furnace atomic absorption spectrometry (HR-CS-GFAAS). This system was designed to use four hollow fibres simultaneously with the same sample, thus improving the simplicity, speed and reproducibility of the results. The organic liquid membrane bis-(2,4,4-trimethylpentyl) phosphinic acid (Cyanex^®^ 272) dissolved in dihexylether (DHE) was immobilised into the pores of the walls of polypropylene hollow fibres. After extraction, the cadmium-enriched acidic phases were recovered and analysed by triplicate. To optimise the extraction process, the effect of both physical and chemical variables was studied, and optimum results with an enrichment factor (EF) of 292 were obtained for a fibre length of 6 cm, 1.06 M Cyanex 272, 0.04 M HNO_3_, stirring rate of 600 rpm and an extraction time of 4.26 h. For practical applications, extraction time was reduced to 2 h, keeping the EF as high as 130. Under these conditions, a detection limit of 0.13 ng L^−1^ Cd(II) was obtained, with a reproducibility of 3.3 % and a linear range up to 3 µg L^−1^ being achieved. The proposed method was successfully applied to the determination of cadmium in mineral, tap and seawater samples.

## 1. Introduction

Sample preparation is still the bottleneck in many analytical procedures and specifically in the determination of trace metals in waters, where very low limits of detection are required for samples with complex matrices (e.g., seawater, estuarine or groundwater). Thus, a sample preparation step is often needed, and liquid membranes have demonstrated to be an excellent tool for this purpose [1,2,3]. Aligned with the principles of sustainability and green chemistry, liquid microextraction-based methods have arisen as a promising alternative for an efficient sample cleanup and/or analyte preconcentration. They have been mostly applied to organic compounds and, in less extension, to the separation of metal species [4,5,6]. Nonetheless, their implementation as routine methods in analytical laboratories has not been fully achieved, so further study and development is needed.

Cadmium is among the most toxic metals in the environment, as reflected in the list of priority pollutants published by the United States Environmental Protection Agency (USEPA) [7] and the European legislation [8,9]. Its presence in aquatic ecosystems can be related to sewage discharge and particles generated from different anthropogenic activities, such as burning of fossil fuels, mining industries, phosphate fertilisers or Ni-Cd batteries, among others [10]. It can be accumulated through the trophic chain and can cause severe damage to human health, even at very low concentrations [11].

Different analytical techniques have been applied for cadmium determination in natural waters [12,13,14,15,16,17,18,19]. Among them, inductively coupled plasma mass spectrometry (ICP-MS) makes it possible to reach the limits of detection usually required for the analysis of trace metals in water samples, but the high cost and the impossibility of analysing saline waters often prevent its use [20]. Moreover, an initial preconcentration step is often needed before the instrumental detection [21]. Therefore, and taking into account that water samples may contain cadmium at concentrations as low as a few ng L^−1^, it is necessary to develop sample-preparation methods for accurate and sensitive determination.

One of the advantages of liquid-phase microextraction (LPME) is the reduction of the solution volumes used for the separation/preconcentration process, allowing it to be considered a green technique [22,23,24]. In fact, only a few microlitres of an extraction solution are frequently used, making graphite furnace atomic absorption spectroscopy (GF-AAS) an attractive and efficient alternative for the quantification step due to its capacity to determine trace metals with very low samples volumes [25,26,27].

In LPME, the analyte is extracted from the sample into a small volume of an organic solution, and then it may be stripped into an appropriate acceptor solution, acting as a liquid membrane system. In this case, both extraction and re-extraction are done in a single step. Although a variety of approaches may be found, three methodologies can be distinguished: Single drop liquid-phase microextraction (SDME), dispersive liquid–liquid microextraction (DLLME) and hollow fibre liquid-phase microextraction (HF-LPME) [6,28]. The application of these systems to the determination of cadmium in environmental waters has also been reviewed [29].

If compared with the others, HF-LPME exhibits higher stability since the extraction solution is located in the pores of a polymeric hollow fibre that separates the two aqueous phases, the donor and the acceptor solutions.

Some approaches to the determination of cadmium in water samples have been previously studied, most of them with non-saline waters and for non-environmental concentrations [30,31,32,33]. Application to seawater is still scarce and mostly focused on the effect of saline matrix and extraction mechanism [33,34,35] and the behaviour and extraction mechanism of chloro-complexes [36,37].

In the present study, the commercial organophosphorus extractant bis-(2,4,4-trimethylpentyl) phosphinic acid (Cyanex^®^ 272), dissolved in dihexylether (DHE), has been used as the organic liquid membrane for an LPME-based method to preconcentrate cadmium from water samples. The effect of the main chemical and physical variables affecting the microextraction system has been studied by applying a dual optimisation methodology, with a first univariate pre-optimisation followed by a multivariate optimisation strategy based on a central composite design. The proposed method was successfully applied to the preconcentration and determination of cadmium at the ng L^−1^ level in mineral, tap and seawater samples.

## 2. Materials and Methods

### 2.1. Reagents and Solutions

All chemicals were reagent grade and they were used as received without further purification. Potassium bromide, sodium sulphate, sodium chloride and tartaric acid were obtained from Merck (Darmstadt, Germany). Tri-sodium citrate 2-hydrate and acetic acid were provided by Panreac (Barcelona, Spain). Nitric acid (trace analysis, 63%) was obtained from Sigma–Aldrich (Steinheim, Germany) and sodium hydroxide from Scharlab (Barcelona, Spain). 2-hydroxyethyl piperazine-N’-(2-ethanesulphonic acid) (HEPES) was supplied by Biochemical (Barcelona, Spain).

Aqueous solutions of cadmium were prepared from a 1000 mg L^−1^ Cd(II) standard solution (Merck) with Milli-Q deionised water (Millipore, Bedford, MA, USA). The organic phases were obtained by dissolving the appropriate amount of bis-(2,4,4-trimethylpentyl) phosphinic acid (Cyanex^®^ 272) from CYTEC (Woodland Park, NJ, USA) in dihexylether from Aldrich (Steinheim, Germany).

### 2.2. Equipment

Polypropylene hollow fibres, Accurel PP S6/2 from 3 M-Membrana (Wuppertal, Germany), with an internal diameter of 1800 µm, 450 µm wall thickness and nominal pore size of 0.2 µm, were used as the support for the liquid membranes. The length of the fibres used varied between 6 and 14 cm, resulting in an internal volume between 150 and 356 µL. The lumen of the hollow fibres was filled and emptied with the strip solution by a Perimax 12 peristaltic pump from Spetec (Erding, Germany), equipped with Tygon tubing while the solution remained stopped during extraction experiments. An Agimatic-SD digital magnetic stirrer from JP Selecta (Barcelona, Spain) was used to perform HF-LPME experiments. A continuum source atomic absorption spectrometer model ContrAA 700 from Analytik Jena (Jena, Germany) was used to determine Cd concentration in the aqueous solutions by applying graphite furnace atomisation (GFAAS). Transversal heating furnaces made in pyrolytic graphite were used, and 20 µL of acceptor solution were injected for each analysis. The measurements were carried out at 228 nm using 300 °C and 1200 °C as the ashing and atomisation temperatures, respectively, and the signals were recorded by adjusting the detector between pixels 98–102. Aqueous solution pH was monitored by a combined glass Ag/AgCl electrode pH-meter from Crison Instruments (Barcelona, Spain).

### 2.3. Extraction Procedure

**Figure 1:**
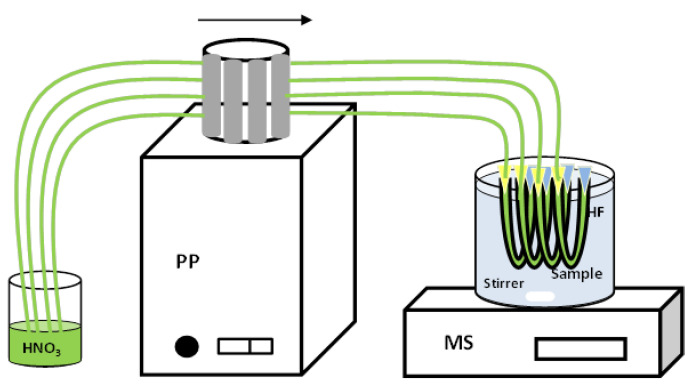
Set-up of the HF-LPME system. PP: Peristaltic pump. MS: Magnetic stirrer. HF: hollow fibre.

For each experiment, four segments of hollow fibres were carefully cut using a blade and then immersed into the Cyanex^®^ 272 in DHE solution for 15 min. The organic solution contained in the pores of each fibre was as low as 31.6 µL, which greatly reduced impact on the environment. After impregnation, the hollow fibres were adjusted into a “U” shape, and connected to a peristaltic pump with corresponding pump tubing to fill the lumens of the fibres with the acceptor HNO_3_ solution (Figure 1). Following this, the pump tubes were disconnected and the extraction process was carried out by stirring the sample at room temperature for a pre-determined time. Finally, the acceptor solution of each fibre was recovered in a different vial, and Cd(II) concentration was measured by GFAAS. This procedure is able to obtain four replicates simultaneously in a single experiment. The efficiency of the process was estimated from the enrichment factor (Equation (1)), with *C_o_* being the initial Cd(II) concentration in the feed solution and *C_f_* being the Cd(II) concentration in the strip solution after extraction.

(1) $$EF=\frac{C_{f}}{C_{o}}$$

### 2.4. Optimisation of the Supported Liquid Membrane Method

As mentioned before, a dual optimisation methodology was used. First, for the univariate pre-optimisation method, the effect of extraction time, fibre length, Cyanex^®^ 272 concentration in the liquid membrane, HNO_3_ concentration in the receiving solution and stirring rate were evaluated. For optimisation studies, samples containing 0.1 µg L^−1^ Cd in NaNO_3_ 0.1 M and buffered at pH = 7.5 with HEPES 0.1 M were used. Taking into account that the proposed method was intended to be used for natural waters, sample pH was always kept at 7.5. In each experiment, the device containing the four hollow fibres was immersed in a vessel with 250 mL of sample and the extraction was carried out.

Subsequently, a multivariate optimisation procedure based on a central composite design was carried out both for better optimisation and in order to correct the effect of possible interactions between the variables. In this case, the variables included were those affected by higher interactions (extraction time, Cyanex^®^ 272 concentration in the organic phase and HNO_3_ concentration in the receiving solution). The initial conditions were selected from the results obtained in the univariate preoptimisation.

In both procedures, the response variable was the EF, and experiments were run in duplicate and randomised to minimise the effect of uncontrolled variables [38].

Software Statgraphics 5.1 from Startpoint Technologies (Addison, TX, USA) was used for all the calculations carried out in the optimisation procedures.

## 3. Results

### 3.1. Univariate Pre-Optimisation of the Supported Liquid Membrane Method

#### 3.1.1. Effect of Extraction Time

**Figure 2:**
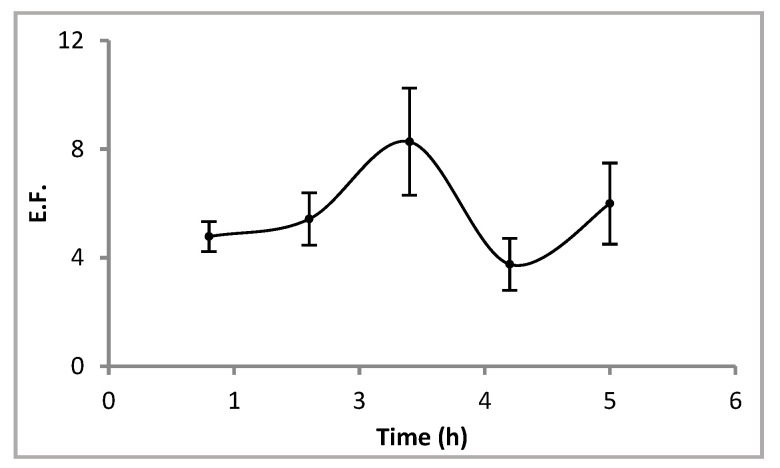
Effect of extraction time on the enrichment factor. Sample: 0.1 µg L^−1^ Cd^2+^ in NaNO_3_ 1 M and HEPES 0.1 M. Liquid membrane: Cyanex^®^ 272 1 M in dihexyl ether. Acceptor solution: HNO_3_ 1 M. Fibre length: 10 cm. Stirring rate: 200 rpm.

All the experiments were carried out with hollow fibres 10 cm long and with stirring of the sample at 200 rpm. The samples were prepared containing 0.1 µg L^−1^ Cd^2+^ in NaNO_3_ 1 M and HEPES 0.1 M. The hollow fibres consisted of Cyanex^®^ 272 1 M in dihexyl ether as liquid membrane impregnating its pores, and HNO_3_ 1 M in the lumen as the acceptor solution. Figure 2 shows the results obtained when extraction time was varied within the range 1–5 h. An increase in EF was obtained in times of up to 3 h. In these systems, the transport of the analyte from the sample to the receiving solution is mainly governed by the diffusion process through the liquid membrane impregnating the pores of the wall of the hollow fibre, which is 450 µm in thickness, and through minimisation of the interfaces processes. Therefore, it is expected that the longer the extraction time, the higher the EF until equilibrium is reached. For higher extraction times, lower EF and higher irreproducibility of the results were obtained, probably due to instability of the organic solution contained into the pores of the fibre. Therefore, the next experiments were carried out at 3 h.

#### 3.1.2. Effect of Hollow Fibre Length

**Figure 3:**
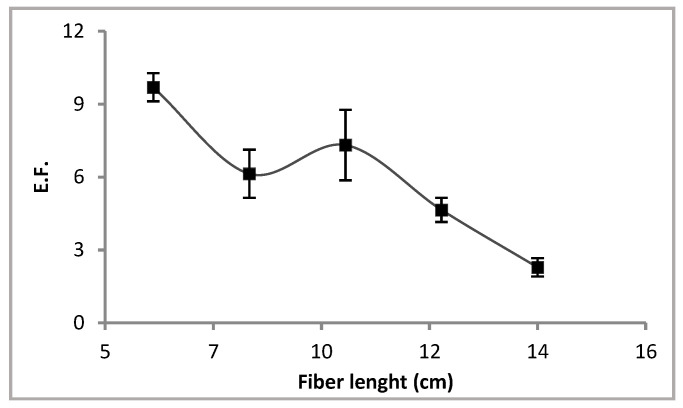
Effect of the fibre length on the enrichment factor. Sample: 0.1 µg L^−1^ Cd^2+^ in NaNO_3_ 1 M and HEPES 0.1 M. Liquid membrane: Cyanex^®^ 272 1 M in dihexyl ether. Acceptor solution: HNO_3_ 1 M. Extraction time: 180 min. Stirring rate: 200 rpm.

The influence of hollow fibre length on the transport of the analyte from the sample through the supported liquid membrane and into the acceptor solution was directly related with the volume of the later in the lumen. An increase in the fibre length implies a greater volume of the acceptor phase and a dilution effect of the extracted analyte, which leads to a decrease in EF. However, when increasing fibre length, a greater contact area between analyte and liquid membrane is obtained and an EF increase is expected. In the proposed method, the fibre length was studied between 6–14 cm and the results showed that EF decreased with an increase in this variable (Figure 3). Therefore, the dilution effect prevailed over the contact area, so a fibre length of 6 cm was selected as optimum for the following experiments.

#### 3.1.3. Effect of Cyanex^®^ 272 Concentration

**Figure 4:**
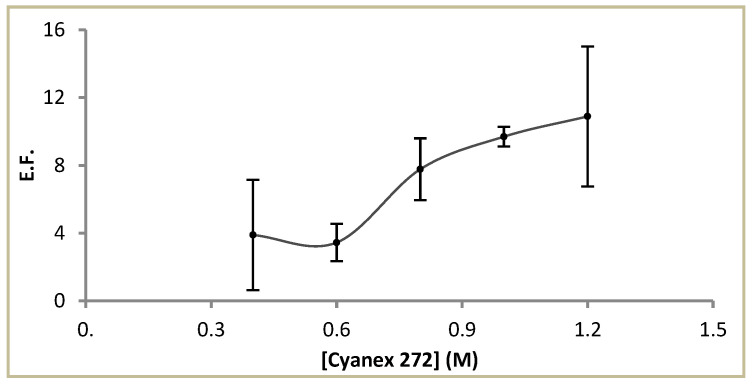
Effect of Cyanex ^®^ 272 concentration on the enrichment factor. Sample: 0.1 µg L^−1^ Cd^2+^ in NaNO_3_ 1 M and HEPES 0.1 M. Acceptor solution: HNO_3_ 1 M. Extraction time: 180 min. Fiber length: 6 cm. Stirring rate: 200 rpm.

The results obtained when Cyanex^®^ 272 concentration was varied from 0.4 M to 1.2 M showed an improvement in the EF up to the higher concentration assayed (Figure 4). However, at this concentration, a higher irreproducibility was observed in the results, probably due to instability of the liquid membrane in the pores of the hollow fibres. This instability was confirmed by the presence of organic droplets in the sample, and is an intrinsic characteristic of supported liquid membranes due to retention of polymeric fibre in the organic phase in the pores by capillary forces [39]. Thus, Cyanex^®^ 272 1 M was selected as the liquid membrane concentration for further experiments.

#### 3.1.4. Effect of HNO_3_ Concentration

**Figure 5:**
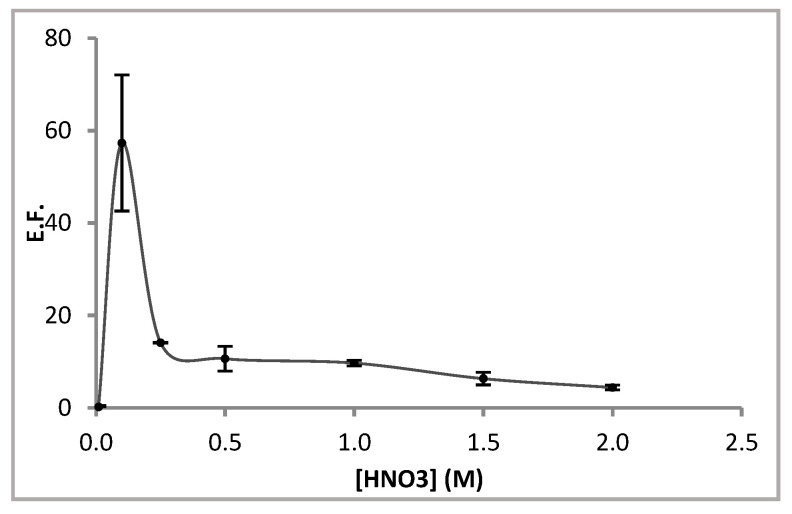
Effect of HNO_3_ concentration on the enrichment factor. Sample: 0.1 µg L^−1^ Cd^2+^ in NaNO_3_ 1 M and HEPES 0.1 M. Liquid membrane: Cyanex^®^ 272 1 M in dihexyl ether. Extraction time: 180 min. Fibre length: 6 cm. Stirring rate: 200 rpm.

Results obtained when the concentration of the acceptor solution varied within the range 0.01–2 M are shown in Figure 5. An improvement in EF was obtained when increasing HNO_3_ concentration up to 0.1 M and poorer EF was obtained for higher concentrations. Thus, much higher acid concentration in the lumen of the fibre could produce instability in the layer between the liquid membrane and acceptor solution, as previously stated by other authors [36]. This instability can be explained in terms of interfacial tension decrease, which has been described for extreme pH values [40]. Therefore, HNO_3_ 0.1 M was selected as the optimum concentration of acceptor solution for the following experiments.

#### 3.1.5. Effect of Stirring Rate

**Figure 6:**
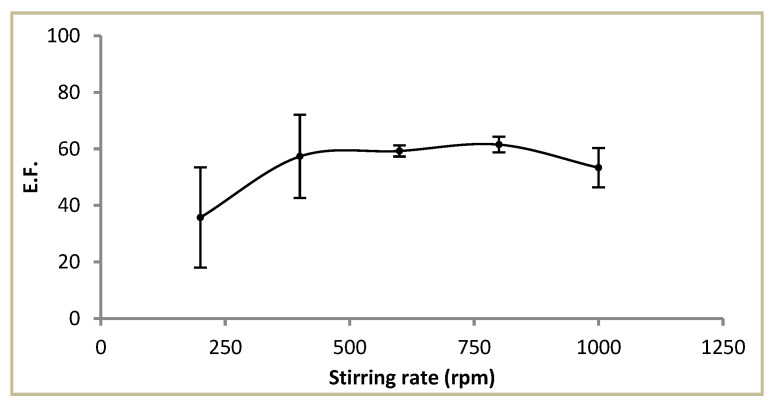
Effect of stirring rate on the enrichment factor. Sample: 0.1 µg L^−1^ Cd^2+^ in NaNO_3_ 1 M and HEPES 0.1 M. Liquid membrane: Cyanex^®^ 272 1 M in dihexyl ether. Extraction time: 180 min. Fibre length: 6 cm. Acceptor solution: HNO_3_ 0.1 M.

The stirring rate is an important factor in HFLPME because the agitation of the sample improves the mass transfer of the analyte and also reaches the equilibrium between the sample solution and the liquid membrane quickly. In the proposed method, it was evaluated between 200–1000 rpm. The results are shown in Figure 6. An increase in the EF was obtained with increasing stirring rate up to 600–800 rpm. The higher rate assayed obtained a decrease in the EF and poorer reproducibility. In these experiments, formation of air bubbles in the surface of the fibres was observed, which has been related to a reduction in the contact area between the sample and the liquid membrane negatively affecting the extraction efficacy [32]. Thus, 600 rpm was selected as the optimum stirring rate for the following experiments.

In summary, after the optimisation of the variables through application of the univariate methodology, the highest EF was obtained when each hollow fibre was 6 cm in length and contained a 1 M Cyanex^®^ 272 in DHE solution as liquid membrane and 0.1 M HNO_3_ as acceptor solution. The best results were obtained at a stirring rate of 600 rpm and an extraction time of 3 h.

### 3.2. Multivariate Optimisation of the Supported Liquid Membrane Method

Based on the information previously obtained in the univariate pre-optimisation, a multivariate optimisation of the microextraction method was carried out and extraction time, Cyanex^®^ 272 concentration and HNO_3_ concentration were evaluated following a central composite design with the surface response methodology. The experimental conditions and the enrichment factor obtained for each of the 32 experiments are shown in Table 1.

An analysis of variance (ANOVA) was carried out in order to evaluate the model and the significance of the effects. In this sense, a second order polynomial equation (Equation (2)) was obtained as the response surface model to fit the experimental data, where t represents the extraction time. The coefficients of determination, R^2^ and adjusted-R^2^, which are a measurement of the amount of the variations around the mean value explained by the model, were 0.9452 and 0.9228, respectively, highlighting its quality.

EF = −176.26 + 46.66·t + 734.01·[HNO_3_] + 332.61·[Cyanex] − 265.23·t·[HNO_3_] + 28.94·t·[Cyanex] − 199.54·[HNO_3_]·[Cyanex]+ 0.86·t^2^ − 2541.77·[HNO_3_]^2^ −177.79·[Cyanex]^2^ (2)

From this expression, the influence of the interactions between variables on the EF may be discussed. In this sense, the negative sign for the interactions nitric acid-time and nitric acid-Cyanex indicates the opposite effect of each individual variable on the response of the system. On the other hand, the interaction time-Cyanex indicates an increase in the EF when both variables take their highest values.

Finally, the maximisation of this function accessed the optimum values of the variables, i.e., t = 4.26 h; [Cyanex 272] = 1.06 M and [HNO_3_] = 0.04 M. Under optimum conditions, an EF value of 292 was obtained.

The optimisation of the function provided a high extraction time, affecting the applicability of the method. For that reason, and taking into consideration that the method provides a very high EF, we selected a compromise value of 2 h for extraction time, a condition under which an EF of 130 was obtained, which is high enough for practical applications.

### 3.3. Selectivity of Cadmium Transport and Chemical Speciation

In order to evaluate the effect of the main ionic species naturally occurring in natural waters on the EF, sample solutions containing 0.1 µg L^−1^ Cd(II) and the potential interfering ion were prepared and analysed by the proposed procedure.

The main cations were used at concentrations levels representative of natural conditions in seawater, i.e., calcium (10.7 mM), potassium (10.6 mM), magnesium (54.8 mM) and sodium (0.5 M). The results showed that the presence of Ca^2+^ and Mg^2+^ had a negative influence on EF, with a decrease of up to 80%. Then, the use of citrate as masking agent was considered. From the results obtained, a 0.01 g L^−1^ solution of the sodium salt of citric acid was shown to be the most appropriate, as previously reported [41]; it was then added for real sample analysis.

**Figure 7:**
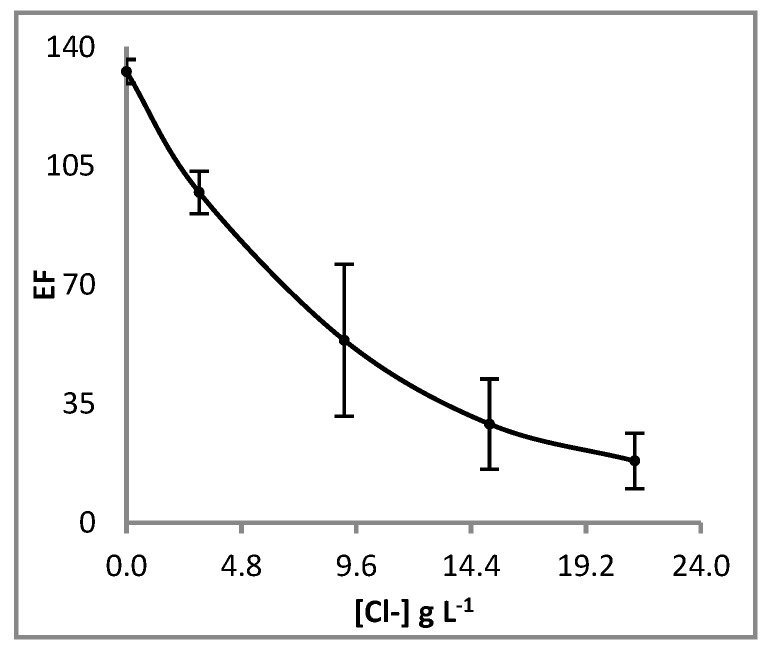
Effect of chloride concentration in the sample on the enrichment factor. Liquid membrane: Cyanex^®^ 272 1.06 M in dihexyl ether. Extraction time: 120 min. Fiber length: 6 cm. Acceptor solution: HNO_3_ 0.04 M.

To evaluate the influence of the main anionic species, chlorides (0.09–0.60 M), bicarbonates (0.1–10 mM), bromides (0.5–10 mM) and sulphates (0.50–4 mM) were added to sample solutions accordingly. From the results it was obtained that only chlorides had a significant effect on the enrichment factor. This behaviour can be explained by the formation of cadmium chloride complexes in the aqueous phase ([CdCl_n_]^(n−2)−^ with n ≥ 2) which could not be transported through the liquid membrane from the sample using Cyanex^®^ 272 at selected conditions. In this case, as shown in Figure 7, the decrease observed in EF should be directly related with the concentration of chloride in the samples, and could open the possibility of using the system for chemical speciation studies. In this sense, increasing chloride concentration in the sample is directly related with the concentration of both free Cd^2+^ and CdCl^+^ in the sample solution, which are the transported species by Cyanex^®^ 272.

Thus, from the results reported above, we obtained a linear relationship between the logarithm of enrichment factor and Cl^−^ concentration (in g L^−1^) in the samples as follows (Equation (3)):

Log (EF) = 2.1003 − 0.0404 [Cl^−^] (3)

Therefore, by measuring the concentration of Cl^−^ in water samples, the estimated EF can be calculated and used for the determination of the total concentration of cadmium in the sample [*Cd*]*_t_* by measuring the concentration of cadmium in the acceptor solution [*Cd*]*_acceptor_* after preconcentration, according to Equation (4).

(4) $${\left[Cd\right]}_{t}=\frac{{\left[Cd\right]}_{acceptor}}{EF}$$

### 3.4. Analytical Performance

To estimate the limit of detection (LOD) of the method, first a calibration set between 0.05 µg L^−1^ Cd and 1 µg L^−1^ Cd and ten independent blank solutions were prepared and analysed by the proposed method. LOD was calculated as the ratio between three times the standard deviation of the blank and the slope of the calibration graph and resulted at 0.13 ng L^−1^ Cd. Similarly, a limit of quantification (LOQ) of 0.34 ng L^−1^ Cd was obtained. As can be seen in Table 2, a very low LOD was obtained even if compared with similar methods based on ICP-MS. From different hollow fibre-based microextraction methods previously reported in the literature, only through employing an ionic liquid as the organic membrane [32] was a detection limit as low as the one obtained here exhibited. The reproducibility obtained was 3.3% (0.1 µg L^−1^ Cd, n = 10). The linearity of the method was evaluated by the quality coefficient (%QC) and by analysing different solutions containing Cd concentration ranging between 0.1 µg L^−1^ and 4 µg L^−1^ by the proposed method. The signal was linear up to 3 µg L^−1^ Cd with a R^2^ of 0.9991.

### 3.5. Application to Real Samples

The proposed method was applied to the determination of Cd in two bottled water samples and a tap water sample from the facilities of the laboratory. Two aliquots of each sample were used. Chloride concentration was measured using a potentiometric probe. One of them was then spiked with 10 ng L^−1^ Cd. After preconcentration experiments, they were analysed and Cd concentration was calculated using Equations (3) and (4). A synthetic seawater sample spiked with three different Cd concentrations was used by using the same procedure. The results obtained, shown in Table 3, proved the applicability of the method to the determination of Cd concentration at the ng L^−1^ level in water samples with different matrices. Additionally, the results obtained for the recovery tests have demonstrated the accuracy of the method for the preconcentration and determination of cadmium in natural waters.

## 4. Conclusions

A very sensitive and environmentally friendly method has been developed for the determination of Cd in natural waters, based on HF-LPME preconcentration and determination by GFAAS. Under optimum conditions, a relationship between the enrichment factor and chloride concentration was obtained, allowing its application in real samples with different salinity.

## Figures and Tables

**Table 1:** Experimental conditions for the central composite design and EF values obtained.

	Time (h)	HNO_3_ (M)	Cyanex^®^ 272 (M)	EF
1	2.25	0.209	0.8	39.26
2	3.5	0.175	1.1	103.16
3	3.5	0.075	0.5	136.94
4	1	0.175	1.1	10.88
5	3.5	0.075	1.1	192.6
6	2.25	0.125	1.3	93.96
7	2.25	0.125	0.8	122.54
8	2.25	0.041	0.8	121.44
9	2.25	0.125	0.8	84.74
10	1	0.175	0.5	13.34
11	4.35	0.125	0.8	231.51
12	1	0.075	0.5	11.82
13	1	0.075	1.1	74.87
14	2.25	0.125	0.3	12.55
15	3.5	0.175	0.5	21.98
16	0.15	0.125	0.8	0.52
17	2.25	0.209	0.8	21.08
18	3.5	0.175	1.1	108.86
19	3.5	0.075	0.5	131.28
20	1	0.175	1.1	13.4
21	3.5	0.075	1.1	196.54
22	2.25	0.125	1.3	106.97
23	2.25	0.125	0.8	99.33
24	2.25	0.041	0.8	146.37
25	2.25	0.125	0.8	69.81
26	1	0.175	0.5	0.8
27	4.35	0.125	0.8	182.94
28	1	0.075	0.5	22.88
29	1	0.075	1.1	70.97
30	2.25	0.125	0.3	5.53
31	3.5	0.175	0.5	16.01
32	0.15	0.125	0.8	0.3

**Table 2:** Comparison of the proposed method with other reported methods.

Technique	Vol. (mL)	EF	LOD (ng L^−1^)	Reference
SAGD-ICP-OES ^a^	10	--	60	16
NH_3_-DRC-ICP-MS ^b^	40	2	5	17
SQ-ICP-MS ^c^	10	30	0.63	18
HFRLM-FAAS ^d^	20	107	1500	30
HFLPME-IL-TS-FF-AAS ^e^	100	90	9	31
HFLPME-IL-GFAAS ^f^	50	162	0.12	32
HFLPME-ETAAS ^g^	25	30	4	34
3SBME-IL-GFAAS ^h^	35	65	4.5	36
HFLPME-GFAAS ^i^	250	130	0.13	This method

^a^ Solution anode glow discharge–Inductively coupled plasma–Optical emission spectroscopy. ^b^ Ammonia–Dynamic reaction cell–Inductively coupled plasma–Mass spectrometry. ^c^ Single quadrupole–Inductively coupled plasma–Mass spectrometry. ^d^ Hollow-fibre renewal liquid membrane–Flame atomic absorption spectrometry. ^e^ Hollow-fibre liquid-phase microextraction–Ionic liquid–Thermospray flame furnace atomic absorption spectrometry. ^f^ Hollow-fibre liquid-phase microextraction–Ionic liquid–Graphite furnace atomic absorption spectrometry. ^g^ Hollow-fibre liquid-phase microextraction–Electrothermal atomic absorption spectrometry. ^h^ Three-phase solvent bar microextraction–Ionic liquid–Graphite furnace atomic absorption spectrometry. ^i^ Hollow-fibre liquid-phase microextraction–Graphite furnace atomic absorption spectrometry.

**Table 3:** Cadmium concentration obtained in different water samples.

Sample	[Cd]_spiked_ ng L^−1^	[Cd]_found_ ng L^−1^	% R
Bottled water 1	---	4.5	
Bottled water 1	10	14.4	99.4
Bottled water 2	---	2.7	
Bottled water 2	10	11.7	92.3
Tap water	---	2.2	
Tap water	10	14.6	119.9
Seawater	50	67	133.8
Seawater	100	104	103.9
Seawater	200	208	103.8

## Data Availability

Not applicable.
